# ﻿Discover hidden taxa of *Erysiphe* section *Erysiphe* fungi (Ascomycota, Erysiphaceae) based on morphology and multilocus phylogeny in China

**DOI:** 10.3897/mycokeys.118.154217

**Published:** 2025-06-04

**Authors:** Zhao-Yang Zhang, Xue-Lian Wu, Xiao-Xue Lv, Tie-Zhi Liu, Dan-Ni Jin, Li Liu, Shuang-Bao Wang, Jing Feng, Tom Hsiang, Yu Li, Shu-Yan Liu

**Affiliations:** 1 Engineering Research Center of Edible and Medicinal Fungi, Ministry of Education, Jilin Agricultural University, No. 2888 Xincheng Street, Changchun 130118, Jilin Province, China; 2 Laboratory of Plant Pathology, College of Plant Protection, Jilin Agricultural University, No. 2888 Xincheng Street, Changchun 130118, Jilin Province, China; 3 College of Chemistry and Life Sciences, Chifeng University, Chifeng 024000, Inner Mongolia Autonomous Region, China; 4 Key Laboratory of Integrated Pest Management on Crops in Northwestern Oasis, Ministry of Agriculture and Rural Affairs, National Plant Protection Scientific Observation and Experiment Station of Korla, Xinjiang Key Laboratory of Agricultural Biosafety, Institute of Plant Protection, Xinjiang Uygur Autonomous Region Academy of Agricultural Sciences, Urumqi 830091, Xinjiang, China; 5 Environmental Sciences, University of Guelph, Guelph, Ontario, N1G 2W1, Canada

**Keywords:** 28S rDNA, IGS, ITS, morphology, new species, powdery mildew, *RPB2*, *TUB*

## Abstract

Erysiphesect.Erysiphe, a taxonomically significant group within the genus *Erysiphe*, is distinguished from other *Erysiphe* sections by its mycelioid chasmothecial appendages. While approximately half of the known species in this section occur in China, our preliminary assessments suggest that a substantial number of cryptic taxa remain undetected. To address this knowledge gap, we conducted a comprehensive phylogeographic survey evaluating 78 specimens collected from 18 provinces (municipalities/autonomous regions) across China. Our integrative approach combined morphological characterization with molecular phylogenetic analyses using five DNA loci: internal transcribed spacer (ITS), 28S rDNA, intergenic spacer (IGS), RNA polymerase II subunit 2 (*RPB2*), and *β-tubulin* (*TUB*). This study led to the discovery of three newly described species (*E.clematidis***sp. nov.** on *Clematis* spp., *E.limoniicola***sp. nov.** on *Limonium* spp., and *E.paeoniae-suffruticosae***sp. nov.** on *Paeonia×suffruticosa* and two newly recorded species from China (*E.malvae* on *Malvapusilla* and *E.punicae* on *Punicagranatum*). Notably, our phylogenetic framework demonstrates that incorporating IGS, *RPB2*, and *TUB* markers substantially enhances species-level resolution and provides critical insights into cryptic speciation within powdery mildews.

## ﻿Introduction

Powdery mildews are a phytopathologically important group of obligate biotrophic fungi, which cause detrimental plant diseases worldwide on more than 10,000 host species of angiosperms (Amano 1986; Braun and Cook 2012). There are more than 900 powdery mildews species worldwide, belonging to 19 genera (Braun and Cook 2012; Kiss et al. 2020). Over the past twenty years, the taxonomic framework of powdery mildews, particularly the definition of the genus *Erysiphe*, has undergone substantial changes. These revisions have been primarily driven by re-examinations of morphological traits and advancements in molecular phylogenetic analyses. Braun (1978) initially proposed dividing the genus *Erysiphe* into three sections: sect. Erysiphe , sect. Golovinomyces , and sect. Galeopsidis . However, more molecular evidence demonstrated that sect. Golovinomyces and sect. Galeopsidis were not closely related to sect. Erysiphe (Heluta 1988; Cook et al. 1997; Saenz and Taylor 1999). As a result, these two sections were reclassified as independent genera, *Neoerysiphe* and *Golovinomyces*, respectively (Braun 1999). A pivotal consolidation occurred through Braun and Takamatsu’s (2000) seminal work, which synonymized *Microsphaera*, *Uncinula*, and allied genera with *Erysiphe*. This taxonomic synthesis resolved long-standing controversies regarding chasmothecial appendage morphology, revealing these diagnostic characters as phylogenetically non-concordant. Subsequent refinements by Liu and Takamatsu (2006) introduced three morphological (rather than phylogenetic) sections: *Erysiphe*, *Microsphaera*, and *Uncinula*. Later, Braun and Cook (2012) reclassified *Californiomyces* and *Typhulochaeta* as additional sections within the genus *Erysiphe*. This classification was based on molecular data and included the previously recognized sections *Erysiphe*, *Microsphaera*, and *Uncinula*. Undoubtedly, the five sections of *Erysiphe* are, nevertheless, significant, and the differences in the shape of chasmothecial appendages are helpful for identification purposes. In its current classification, the genus *Erysiphe* encompasses more than 50% of the species within the family Erysiphaceae. Approximately 450 different species of Erysiphe in total, with 117 of them belonging to sect. Erysiphe. Nearly half of these species are found in China and are capable of infecting approximately 260 plant species (Braun and Cook 2012; Meeboon et al. 2021; Bradshaw et al. 2022a; Liu et al. 2022b; Darsaraei et al. 2023; Bradshaw et al. 2024c).

The internal transcribed spacer (ITS1-5.8S-ITS2) region and the 28S rRNA gene are standard DNA barcode markers for powdery mildews. Braun and Cook (2012) re-evaluated the structure of the family Erysiphaceae based on phylogenetic results and provided a worldwide taxonomic survey. Meanwhile, rDNA sequences have been widely used for phylogenetic analyses and identification purposes in most genera and species (Takamatsu et al. 2010; Qiu et al. 2019; Bradshaw et al. 2020a; Kiss et al. 2020; Jin et al. 2021; Feng et al. 2022; Liu et al. 2022a; Darsaraei et al. 2023), including the genus *Erysiphe* sections *Microsphaera* and *Uncinula* (Takamatsu et al. 2015a, b). However, ITS analyses often lack resolution on species level in phylogenetic trees, above all in phylogenetically young lineages of powdery mildews with still ongoing speciation processes, such as the *E.aquilegiae* complex (Bradshaw et al. 2020b; Bradshaw et al. 2023c), the *E.alphitoides* complex (Bradshaw et al. 2025), and the *E.trifoliorum* complex (Bradshaw et al. 2020b), which subsequently led to attempts to search for additional markers applicable for phylogenetic analyses of powdery mildews. Ellingham et al. (2019) successfully tested the applicability of seven genes (*actin*, *β-tubulin*, *calmodulin*, *Chs*, *EF1-α*, *MCM7*, and *Tsr1*) in powdery mildews for discrimination purposes at species level. These were useful for improved resolution in critical groups of *Erysiphe* spp. Qiu et al. (2020) conducted a phylogenetic revision of a species complex within the genus *Golovinomyces* using sequences from the ITS, 28S, IGS, *TUB*, and *CHS1* regions. Shirouzu et al. (2020) provided a phylogenetic overview of the Erysiphaceae using nrDNA and *MCM7* sequences. Bradshaw et al. enhanced the phylogenetic analysis of powdery mildews in the family Erysiphaceae, the genera *Cystotheca*, *Golovinomyces*, and *Erysiphe* (the “*Uncinula* lineage” and “*Microsphaera* lineage”), by evaluating additional loci such as *CAM*, *GAPDH*, *GS*, *RPB2*, and *TUB* with the ITS+28S rDNA sequences (Bradshaw et al. 2022b; Bradshaw et al. 2022c; Bradshaw et al. 2023a; Bradshaw et al. 2023b; Bradshaw et al. 2023c; Bradshaw et al. 2024a; Bradshaw et al. 2024b). The incorporation of these multiple loci has proven useful for improving the resolution of critical groups of powdery mildew species.

To uncover more potential species within sect. Erysiphe , we collected and examined 32 specimens from 9 host plant families spanning across 12 provinces (municipalities/autonomous regions) in China. The identification and description of the hidden species were achieved by examining their microscopic morphological features and performing phylogenetic analyses. Additional DNA markers, IGS, *RPB2*, and *TUB*, were involved in the phylogenetic tree construction because ITS and 28S rDNA sequences often lack the necessary resolution to adequately distinguish between certain lineages. The objective of this study is to introduce more novel taxa, augment the species diversity of powdery mildew fungi, and establish a foundation for future phylogenetic and taxonomic research on the genus *Erysiphe*.

## ﻿Materials and methods

### ﻿Sample collection

78 specimens of host plants from 23 families were examined, of which 63 specimens were stored in the Herbarium of Mycology of Jilin Agricultural University (HMJAU), the other 13 specimens were from the Mycological Herbarium of Chifeng University (CFSZ) in Chifeng, and 2 specimens were borrowed from the Herbarium Mycologicum Academiae Sinicae (HMAS) in Beijing. Details of these specimens are provided in Table 1.

**Table 1.** List of taxa, host, voucher, collection province, and GenBank accession number of the specimens examined in this study.

Taxon	Host	Voucher	Collection province	ITS+28S	IGS	*RPB2*	*TUB*
*Erysiphebuhrii*	*Dianthuschinensis*	HMJAU-PM92238	Xinjiang	Pp968516	Pp971986	–	–
*Erysiphebuhrii*	*Dianthuschinensis*	HMJAU-PM92239	Jilin	Pp968517	Pp971987	–	–
***Erysipheclematidis***	***Clematismacropetala***	**HMJAU-PM92226 ^†, ‡^**	**Qinghai**	**Pp968504**	**Pp971974**	–	–
***Erysipheclematidis***	***Clematisglauca***	**HMJAU-PM92225 ^†^**	**Qinghai**	**Pp968503**	**Pp971973**	**Pq867668**	**Pq857975**
***Erysipheclematidis***	***Clematisintricata***	**HMJAU-PM92223 ^†^**	**Qinghai**	**Pp968501**	**Pp971971**	**Pq867666**	**Pq857973**
***Erysipheclematidis***	***Clematisintricata***	**HMJAU-PM92222 ^†^**	**Shaanxi**	**Pp968500**	**Pp971970**	–	–
***Erysipheclematidis***	***Clematisintricata***	**HMJAU-PM92224 ^†^**	**Gansu**	**Pp968502**	**Pp971972**	**Pq867667**	**Pq857974**
***Erysipheclematidis***	***Clematisglauca***	**HMJAU-PM92227 ^†^**	**Gansu**	**Pp968505**	**Pp971975**	**Pq867669**	**Pq857976**
Erysiphediervillaevar.diervillae	*Weigelaflorida*	CFSZ 50307	Inner Mongolia	Pp968459	Pp975483	Pq867642	–
Erysiphediervillaevar.diervillae	*Weigelaflorida*	CFSZ 50033	Inner Mongolia	Pp968460	Pp975484	Pq867641	–
*Erysipheglycines*	*Glycinesoja*	HMJAU-PM92270	Beijing	Pp968548	Pp972018	Pq867698	–
***Erysiphelimoniicola***	***Limoniumbicolor***	**CFSZ 1870 ^§, ‡^**	**Inner Mongolia**	**Pp968457**	**Pp975481**	**Pq867636**	**Pq857963**
***Erysiphelimoniicola***	***Limoniumaureum***	**CFSZ 6794 ^§^**	**Inner Mongolia**	**Pp968458**	**Pp975482**	**Pq867637**	**Pq857964**
*Erysiphemalvae*	*Malvapusilla*	HMJAU-PM92233	Yunnan	Pp968511	Pp971981	–	Pq857984
*Erysiphemalvae*	*Malvapusilla*	HMJAU-PM92234	Yunnan	Pp968512	Pp971982	Pq867677	–
*Erysiphepaeoniae*	*Paeonialactiflora*	HMJAU-PM92258	Liaoning	Pp968536	Pp972006	Pq867692	Pq858000
*Erysiphepaeoniae*	*Paeonialactiflora*	HMJAU-PM92259	Inner Mongolia	Pp968537	Pp972007	–	Pq858001
*Erysiphepaeoniae*	*Paeonialactiflora*	HMJAU-PM92260	Jilin	Pp968538	Pp972008	–	–
*Erysiphepaeoniae*	*Paeonialactiflora*	HMJAU-PM92261	Jilin	Pp968539	Pp972009	–	–
*Erysiphepaeoniae*	*Paeonialactiflora*	HMJAU-PM92262	Hebei	Pp968540	Pp972010	Pq867693	Pq858002
***Erysiphepaeoniae-suffruticosae***	***Paeoniasuffruticosa***	**HMJAU-PM92254 ^§^**	**Gansu**	**Pp968532**	**Pp972002**	**Pq867694**	**Pq858003**
***Erysiphepaeoniae-suffruticosae***	***Paeoniasuffruticosa***	**HMJAU-PM92255 ^§, ‡^**	**Shaanxi**	**Pp968533**	**Pp972003**	**Pq867695**	**Pq858004**
***Erysiphepaeoniae-suffruticosae***	***Paeoniasuffruticosa***	**HMJAU-PM92256 ^§^**	**Gansu**	**Pp968534**	**Pp972004**	**Pq867696**	**Pq858005**
***Erysiphepaeoniae-suffruticosae***	***Paeoniasuffruticosa***	**HMJAU-PM92257 ^§^**	**Jilin**	**Pp968535**	**Pp972005**	**Pq867697**	**Pq858006**
*Erysiphepunicae*	*Punicagranatum*	HMJAU-PM92219	Yunnan	Pp968497	Pp971967	Pq867665	Pq857970
*Erysiphepunicae*	*Punicagranatum*	HMJAU-PM92220	Yunnan	Pp968498	Pp971968	–	Pq857971
*Erysipherumicicola*	*Rumexacetosa*	HMJAU-PM92240	Xinjiang	Pp968518	Pp971988	Pq867680	Pq857987
*Erysipherumicicola*	*Rumexacetosa*	HMJAU-PM92241	Xinjiang	Pp968519	Pp971989	Pq867683	Pq857990
*Erysipherumicicola*	*Rumexacetosa*	HMJAU-PM92242	Gansu	Pp968520	Pp971990	Pq867681	Pq857988
*Erysipherumicicola*	*Rumexacetosa*	HMJAU-PM92243	Xinjiang	Pp968521	Pp971991	Pq867682	Pq857989
*Erysipherumicicola*	*Rumexacetosa*	HMJAU-PM92512	Xizang	Pq871391	Pq870815	–	–
*Erysipherumicicola*	*Rumexacetosa*	HMJAU-PM92513	Xizang	Pq871392	Pq870816	–	–

^†^ Sexual morph; ^‡^ Holotype; ^§^ Asexual morph & Sexual morph; New species were shown in bold.

### ﻿Morphological observations

Fresh leaves with typical symptoms of powdery mildews were used for morphological examination. Chasmothecia were stripped off from the leaf surface with a clean needle, mounted on a microscope slide and examined in 3% (w/v) NaOH using light microscopy (ZEISS ScopeA1, Germany). To examine the asexual morph, hyphae, conidiophores, and conidia of fresh collections were stripped off from the leaf surfaces with clear adhesive tape, mounted on a microscope slide with the fungal mycelium uppermost, and examined in water (Shin and La 1993). Data for morphological characteristics were collected based on more than 30 measurements of the structures concerned.

### ﻿DNA extraction and sequencing

Genomic DNA was extracted by the Chelex-100 method from mycelia and chasmothecia (Walsh et al. 1991; Hirata and Takamatsu 1996), followed by PCR amplification of five genomic regions: the ITS (including 5.8S rDNA) with primers AITS/PM2, PM1/PM2, and ITS4/ITS5 (Takamatsu and Kano 2001; Bradshaw and Tobin 2020); 28S rDNA (D1-D2 domains) using PM3/TW14 and LSU1/LSU2; IGS with IGS-12a/NS1R (Carbone and Kohn 1999); *RPB2* amplified by PMRpb2_4/PMRpb2_6R and ERPB2_3/ERPB2_7R; and *TUB* using BTF5b/BTR7a and ETUB2/ETUB2R (Ellingham et al. 2019; Bradshaw et al. 2022c, 2023a). Reactions (25 μL total volume) contained 2 μL template DNA, 12.5 µL Premix Taq [TaKaRa Taq 1.25 U/25 μL, 2× dNTP Mixture (0.4 mM each), 2× Taq Buffer (3 mM Mg^2+^)] (TaKaRa, Tokyo, Japan), 1 μL each primer (20 ng/μL), and ddH_2_O, cycled through initial denaturation (95 °C for 5 min), 35 cycles of 94 °C for 1 min, 52–58 °C for 30 s, and 72 °C for 1 min, with final extension at 72 °C for 10 min, including template-free negative controls. Amplification success was verified through 1.2% agarose gel electrophoresis (0.5× TBE), followed by bidirectional Sanger sequencing (Sangon Biotech, Shanghai) using original primers. Sequences were assembled in DNAMAN 6.0 and base-corrected using BIOEDIT 7.0.

### ﻿Phylogenetic analysis

Multiple sequence alignments were conducted by MUSCLE implemented in MEGA-X, including those retrieved from GenBank, with ambiguous regions manually adjusted (Kumar et al. 2018). Alignments were further manually refined and deposited in TreeBASE (https://www.treebase.org/) under the accession number 32032 (ITS+28S) and 32024 (ITS+28S+IGS+*RPB2*+*TUB*). Phylogenetic analyses employed three complementary approaches: maximum parsimony (MP) in PAUP 4.0 (Swofford 2002) identified optimal trees through heuristic searches (100 replicates), calculating tree statistics including consistency (CI), retention (RI), and rescaled consistency (RC) indices; Bayesian inference (BI) in MrBayes 3.2.7 (Ronquist et al. 2012) utilized models selected via MrModeltest v.2 (Nylander 2004), running 1,000,000 generations with sampling every 1,000 steps and 25% burn-in discard, terminating when split frequency standard deviations stabilized below 0.01; and maximum likelihood (ML) in RAxML v.2.0.10 (Edler et al. 2020) implemented GTRGAMMA with 1,000 bootstrap replicates (Felsenstein 1985). All methods produced congruent topologies, with horizontal branch lengths in the selected MP tree reflecting inferred substitutions. Bootstrap supports from BI and ML analyses were superimposed on the MP framework, using *E.glycines* (Takamatsu et al. 2015a) as the designated outgroup.

### ﻿Abbreviations

**BI** Bayesian inference

**BS** bootstrap support

***CAM*** calmodulin

**CFSZ** Chifeng College, Mycological Herbarium

**Chs** chitin synthase

**CI** consistency index

**DNA** Deoxyribonucleic acid

***EF1-α*** elongation factor 1 alpha

***GAPDH*** glycer-aldehyde-3-phosphate dehydrogenase

***GS*** glutamine synthetas

**HMAS** Herbarium Mycologicum Academiae Sinicae

**HMJAU** Herbarium of Mycology of Jilin Agricultural University

**IGS** intergenic spacer

**ITS** Internal Transcribed Spacer

**LSU** The nuclear ribosomal large subunit

***MCM7*** Minichromosome maintenance proteins 7

**ML** Maximum likelihood

**MP** Maximum parsimony

**nrDNA** Ribosomal RNA Gene

**PP** Posterior probabilities

**RC** rescaled consistency index

**RI** retention index

***RPB2*** The second largest subunits of RNA polymerase II

**PCR** Polymerase chain reaction

**rRNA** ribosomal RNA

**s. lat.** sensu lato

**s. str.** sensu stricto

**TL** Tree length

**Tsr1** Ribosome Maturation Factor

***TUB*** β-tubulin

## ﻿Results

### ﻿Phylogenetic analysis

**Figure 1.**
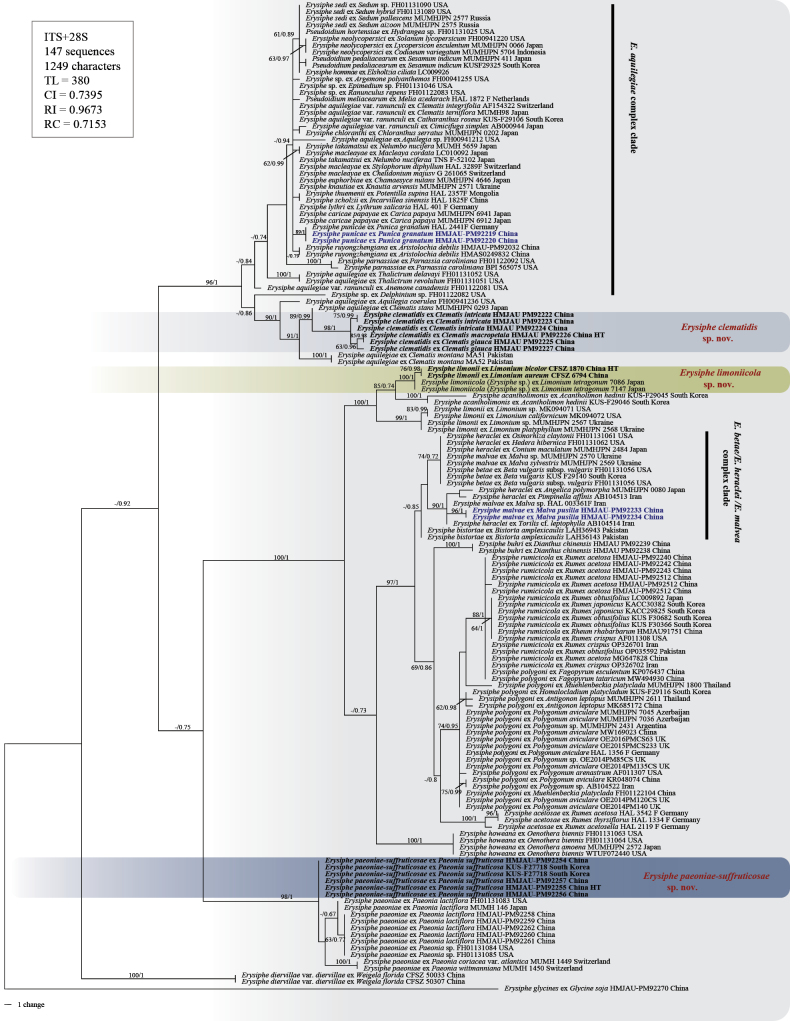
Phylogenetic tree of selected species within sect. Erysiphe based on the ITS+28S regions. Bootstrap values greater than 60% for the maximum likelihood (ML) analyses are displayed followed by posterior probabilities ≥ 0.70 of Bayesian inference (BI) analyses. HT = Holotype; the color box indicates new species; bold font indicates the new species obtained in this study; blue bold font indicates new record species.

**Figure 2.**
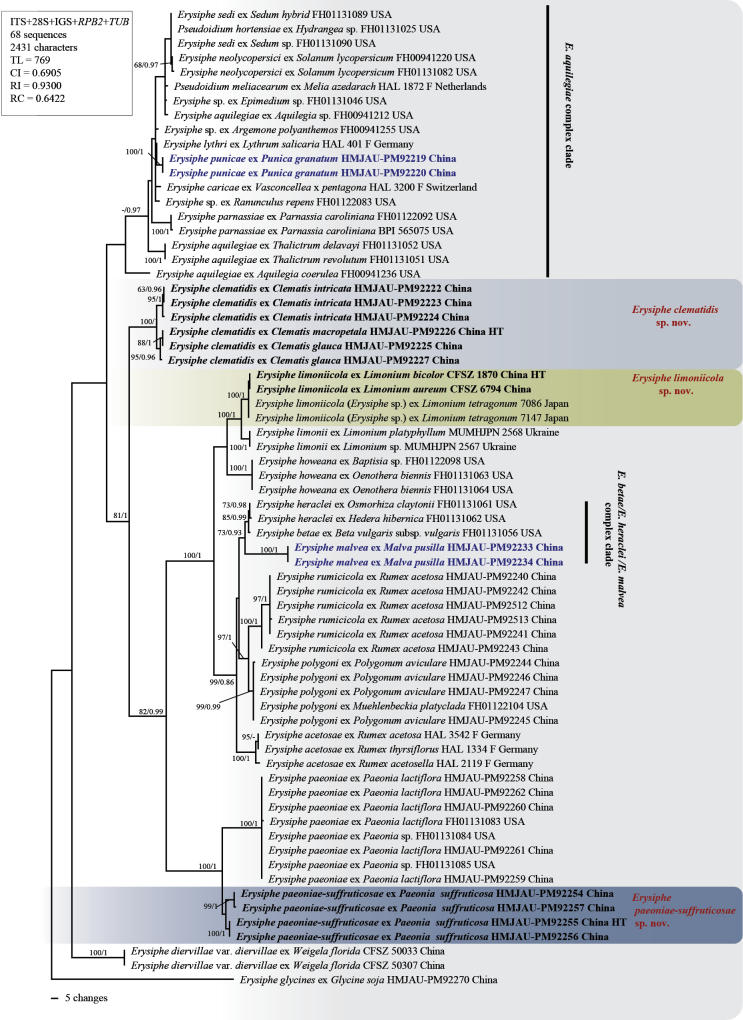
Phylogenetic tree of selected species within sect. Erysiphe based on the ITS+28S+IGS+*RPB2*+*TUB* regions. Bootstrap values greater than 60% for the maximum likelihood (ML) analyses are displayed followed by posterior probabilities ≥ 0.70 of Bayesian inference (BI) analyses. HT = Holotype; the color box indicates new species; bold font indicates the new species obtained in this study; blue bold font indicates new record species.

A total of 32 ITS+28S rDNA, 32 IGS, 21 *RPB2*, and 20 *TUB* regions sequences were obtained in this study and were deposited in GenBank (Table 1). They were used in the phylogenetic analyses, incorporating sequences of closely related species of sect. Erysiphe retrieved from GenBank (Suppl. material 2). The phylogenetic trees unveiled the molecular relationships among these species (Figs 1, 2).

While ITS+28S data could distinguish most species, there was sometimes insufficient genetic differentiation for different species parasitizing the same host family, such as *E.paeoniae-suffruticosae* sp. nov. and *E.paeoniae* on Paeoniaceae (Fig. 1). ITS+28S+IGS and multilocus (ITS+28S+IGS+*RPB2*+*TUB*) phylogenetic trees showed that three putative novel species formed clearly separated clades from the previously sequenced taxa with high support (Fig. 2, Suppl. material 1). *E.clematidis* sp. nov. was clustered next to the *E.aquilegiae* complex clade; *E.limoniicola* sp. nov. clusters with *E.limonii* and *E.acantholimonis*, both of which also parasitize plants in the Plumbaginaceae; and *E.paeoniae-suffruticosae* sp. nov. grouped with *E.paeoniae*, a species associated with the Paeoniaceae. Additionally, the two new record species, *E.malvae* on *Malvapusilla* and *E.punicae* on *Punicagranatum*, were marked in blue font in the phylogenetic trees. These molecular phylogenetic analyses provide strong support for the delimitation of the new species.

### ﻿Taxonomy

#### ﻿ Erysiphe clematidis

Taxon classificationFungiHelotialesErysiphaceae

Zhao Y. Zhang & S.Y. Liu sp. nov.

EB51B541-4A77-514E-BDB1-F272A0E33027

857028

Fig. 3

##### Etymology.

Epithet derived from the name of the host genus, *Clematis*.

**Figure 3.**
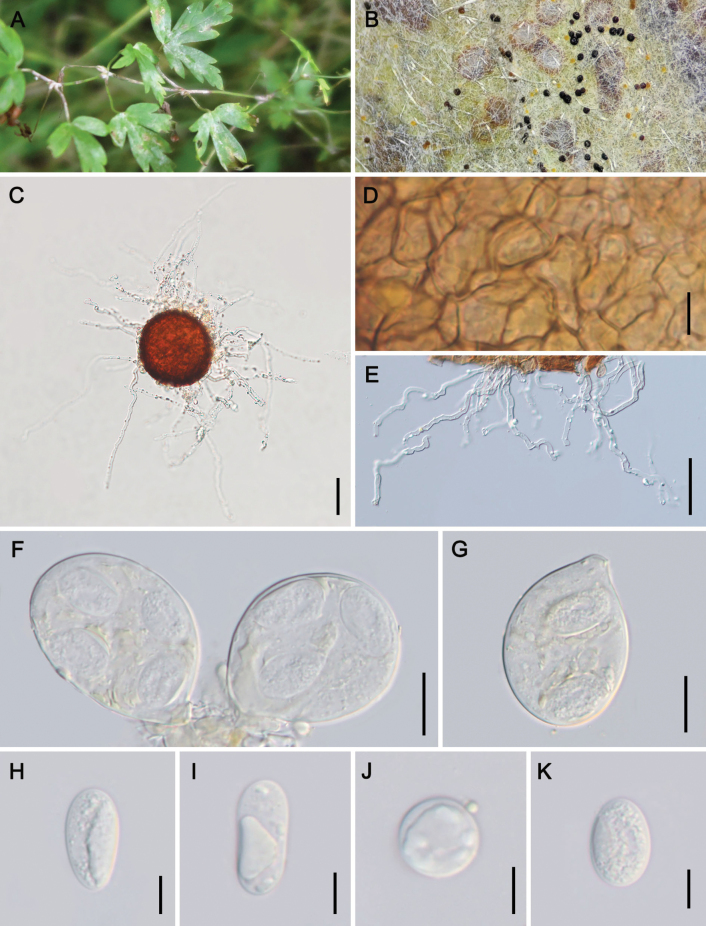
Symptoms and sexual morph of *Erysipheclematidis* sp. nov. on *Clematismacropetala* (holotype: HMJAU-PM92226) **A, B** powdery layer and chasmothecia on leaves **C** chasmothecium **D** peridium cells **E** appendages **F, G** asci **H–K** ascospores Scale bars: 10 µm (**D, H–K**); 50 µm (**C, E**); 20 µm (**F, G**).

##### Diagnosis.

Morphologically resembling E.aquilegiaevar.ranunculi, but distinguished by chasmothecial appendages that are colorless or only pigmented at the base, only with 0–2 septa. Differs from var. aquilegiae in having much shorter appendages, 0.7–3.5 times as long as the chasmothecial diam., mycelioid, geniculate-sinuous, and colorless or only pigmented at the base. Furthermore, forming a highly supported species clade in all phylogenetic analyses, in sister position to the *E.aquilegiae* cluster.

##### Description.

Mycelium on leaves and stems, amphigenous, effuse or in patches, on stems colonies usually thick, ± persistent. **Sexual morph**: Chasmothecia mostly scattered, dark brown, mainly on leaf abaxial surface of leaves and stems, more abundant on the upper side of the leaf blade, (66–)74–122(–129) µm; peridium cells irregularly polygonal, 5–13 µm; appendages unequal in number, mycelioid, continuously curved, with small irregular branchlets, some bifurcate branches present, 0–2-septate, hyaline, sometimes yellowish brown near the base, or light brown, paler or colorless towards the apex, uneven in thickness, length about 0.7–8.6 times as long as the chasmothecial diam., sometimes up to 338 μm; asci 4–10, (32–)46–73 × 27–50 µm, ellipsoid-obovoid, short-stalked to sessile; ascospores (1–)2–4, 16–28 × 13–19 µm, subglobose, ovoid, irregularly ovoid, ellipsoid, reniform, colorless.

##### Holotype.

China, Qinghai Prov. • 1; Xining City; 36°38'2"n, 101°45'36"e; ca. alt. 2260 m a.s.l.; 15 Oct. 2023; Zhao-Yang Zhang & Li Liu leg.; on *Clematismacropetala*; HMJAU-PM92226. *Isotype*: same data as for holotype; HMAS 353407.

##### Distribution.

Asia (China).

##### Host.

*Clematis* spp. (Ranunculaceae).

##### Additional material examined.

China, Gansu Prov. • 1; Dingxi City; 36°36'33"n, 104°35'5"e; ca. 1860 m a.s.l.; 17 Oct. 2023; Zhao-Yang Zhang & Li Liu leg.; on *Clematisglauca*; HMJAU-PM92227. • 1; Zhangye City; 38°55'56"n, 100°23'45"e; ca. 1456 m a.s.l.; 23 Oct. 2023; Dan-Ni Jin & Xue-Lian Wu; on *Cl.intricata*; HMJAU-PM92224. –Qinghai Prov. • 1; Xining City; 36°37'17"n, 101°47'7"e; ca. 2278 m a.s.l.; 12 Oct. 2023; Zhao-Yang Zhang & Li Liu leg.; on *Cl.glauca*; HMJAU-PM92225. • 1; Xining City; 36°37'17"n, 101°47'7"e; ca. 2280 m a.s.l.; 12 Oct. 2023; Zhao-Yang Zhang & Li Liu leg.; on *Cl.intricata*; HMJAU-PM92223. –Shaanxi Prov. • 1; Yulin City; 38°15'14"n, 109°44'51"e; ca. 1040 m a.s.l.; 18 Oct. 2023; Zhao-Yang Zhang & Li Liu leg.; on *Cl.intricata*; HMJAU-PM92222.

##### Notes.

**Figure 4.**
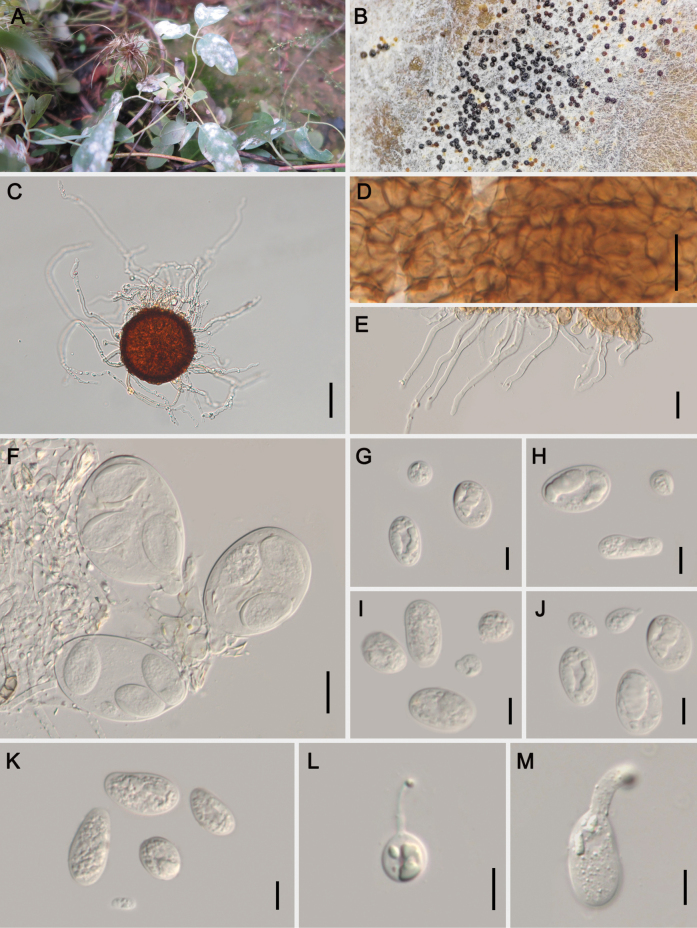
Symptoms and sexual morph of *Erysipheclematidis* sp. nov. on *Clematisglauca* (HMJAU-PM92227) **A, B** powdery layer and chasmothecia on leaves **C** chasmothecium **D** peridium cells **E** appendages **F** asci **G–K** two types of ascospores **L, M** germinating ascospores Scale bars: 50 µm (**C**); 20 µm (**D–F**); 10 µm (**G–M**).

Based on the applied morphological concept of *Erysipheaquilegiae*s. lat., Braun and Cook (2012) listed Clematis spp. as hosts of var. aquilegiae as well as var. ranunculi. In this study, we assigned the new species *Erysipheclematidis* for our collections on genus *Clematis* based on the obvious morphological differences. The appendages of *E.clematidis* are significantly shorter than those of E.aquilegiaevar.aquilegiae [0.7–8 vs. (1–)3–12 times as long as the chasmothecial diam.], and the color of their appendages also differs: the appendages of *E.clematidis* have basal pigmentation (colorless to faintly pigmented), whereas those of E.aquilegiaevar.ranunculi are brown throughout or paler towards the apex. Unexpectedly, two types of ascospore morphologies were observed on the specimen of *C.glauca* (HMJAU-PM92227): macro-ascospores [14–31 × (6–)10–18 µm] and micro-ascospores [4–17 × 4–14 µm] (Fig. 4). Furthermore, some germinating ascospores were also observed, which is very unusual in powdery mildews. More specimens need to be collected to determine whether this phenomenon is common.

#### ﻿ Erysiphe limoniicola

Taxon classificationFungiHelotialesErysiphaceae

Zhao Y. Zhang, S.Y. Liu & T.Z. Liu sp. nov.

F8B6FE8C-2ED7-508C-ABD8-2A6C851ED4D7

857032

Fig. 5

##### Etymology.

Epithet derived from the name of the host genus, *Limonium*, + cola (dweller).

**Figure 5.**
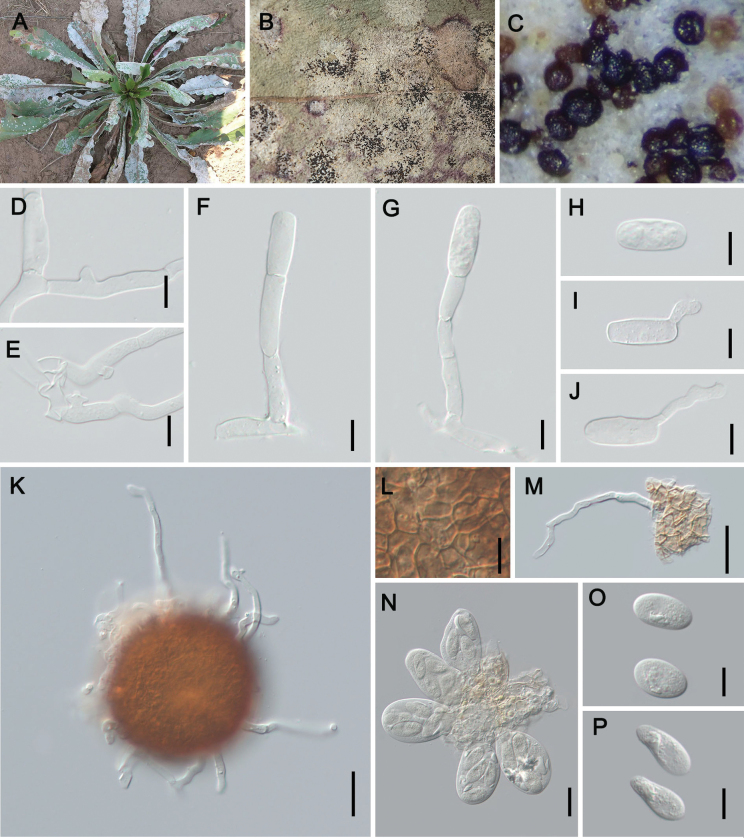
Symptoms and morphology of *Erysiphelimoniicola* sp. nov. on *Limoniumbicolor* (holotype: CFSZ 8906) **A–C** powdery layer and chasmothecia on leaves **D, E** hyphal appressoria **F, G** conidiophores **H** conidium **I, J** germ tubes **K** chasmothecium **L** peridium cells **M** appendage **N** asci **O, P** ascospores. Scale bars: 10 µm (**D–J, M, O, P**); 20 µm (**N**); 25 µm (**K, L**).

##### Diagnosis.

Differs from *Erysipheaurea* and *E.limonii* on hosts of the genus *Limonium* in the size of the chasmothecia, 69–112 μm diam., the number of asci, (3–)4–9, and the number of septa in the appendages, 0–2(–4), variably shaped ascospores, and by forming a strongly supported species clade in phylogenetic analyses.

##### Description.

Mycelium amphigenous, forming dense, thin to thick, persistent patches or complete covers; hyphae 3–6 μm wide, septate, straight or curved, smooth; hyphal appressoria solitary or occasionally in opposite pairs, nipple-shaped or lobed. **Asexual morph**: Conidiophores arising from superficial hyphal mother cells, erect, straight or curved, 48–103 × (5–)6–8 μm (without conidia), foot cells subcylindrical, some distinctly curved at the base, the whole foot cell sometimes wavy-curved, 20–42 × 5–7 μm, the basal septum is mostly elevated, up to 10 µm, followed by 1–3 shorter cells of about the same length or shorter; conidia single, narrowly cylindrical, 21–39 × 10–17 μm; germ tubes near the shoulder, with septa, short to medium in length, showing longitubus pattern, conidial appressoria unlobed but enlarged or curved near the top. **Sexual morph**: Chasmothecia gregarious, 69–112 μm diam.; peridium cells irregularly polygonal, 4–15 μm diam.; appendages few to many, up to 28, often interwoven with the mycelium, unbranched, mycelium-like, irregularly curved or bent, the appendages are about as long as the chasmothecial diam. or shorter, width uneven, tapering at the tip, which is often obtuse-acuminate, 4–8 µm wide, 0–2(–4)-septate, walls thin, smooth to rough, colorless; asci (3–)4–9, ellipsoid, ovoid, broadly ovoid, distinctly long-stalked or subsessile, about 47–88 × 30–49 μm; ascospores 3–5, broadly ellipsoid-ovoid, oblong-ovoid, sole-shaped, 17–30 × 8–21 μm.

##### Holotype.

China, Inner Mongolia Autonomous Region • 1; Xilinhot City; 43°57'23"n, 116°6'46"e; ca. 1100 m a.s.l.; 28 Sep. 2009; Tie-Zhi Liu & M. Chen leg.; on *Limoniumbicolor*; CFSZ 1870. Isotype: same data as for holotype; HMAS 353411.

##### Host.

*Limonium* (*bicolor*, *aureum* and *tetragonum*) (Plumbaginaceae).

##### Distribution.

Asia (China and Japan).

##### Additional material examined.

China, Inner Mongolia Autonomous Region • 1; Sonid Left Banner; 43°51'21"n, 113°38'56"e; ca. 1230 m a.s.l.; 11 Sep. 2013; Tie-Zhi Liu leg.; on *L.aureum*; CFSZ 6794.

##### Notes.

The genus *Limonium* (Plumbaginaceae) comprises approximately 600 globally distributed species (Koutroumpa et al. 2018). Within this genus, two powdery mildew species have been described: *Erysipheaurea* and *E.limonii* (Braun and Cook 2012). *E.aurea* is known exclusively from its type collection (HMAS 38952) on *L.suffruticosum* in Xinjiang, China (Zheng and Yu 1987). Previous identifications of powdery mildew on *L.bicolor* and *L.aureum* from Inner Mongolia (CFSZ 1870 and 6794) were attributed to *E.limonii* (Liu 2010, 2022). Comparative morphological analysis of the examined specimens (with specimens CFSZ 1870 and 6794 borrowed from the Mycological Herbarium of Chifeng College) demonstrated clear differentiation from *E.limonii*: chasmothecia were significantly smaller (69–112 vs. 90–180 µm), and appendages exhibited fewer septa [0–2(–4) vs. 0–7]. Furthermore, the two species exhibit variable ascospore shapes, and are distinct from *E.aurea* in having septate appendages and lacking the golden-yellow cells of the inner peridial wall.

Phylogenetic analysis of ITS+28S rDNA sequences revealed that the Inner Mongolian isolates formed a well-supported clade with *Erysiphe* sp. on *L.tetragonum* from Japan (Meeboon et al. 2021), diverging from *E.limonii*. Although a single nucleotide difference in the 28S region was observed, this may reflect geographic or host-associated genetic variation rather than distinct species boundaries. It seems that these two Japanese specimens (= *Erysiphe* sp.) pertain to the new species, *E.limoniicola*. Comparative morphometric analysis further revealed incongruences: while Meeboon et al. (2021) reported conidiophore lengths of 50–127 µm on *L.tetragonum*, our measurements (48–102 µm) partially overlapped with previous records (64–96 µm) (Liu 2010, 2022). Such variability in conidiophore length is a common phenomenon in powdery mildew anamorphs, likely often influenced by external influences.

#### ﻿ Erysiphe paeoniae-suffruticosae

Taxon classificationFungiHelotialesErysiphaceae

Zhao Y. Zhang & S.Y. Liu sp. nov.

73D015CE-5952-5B59-9355-793E352C24E3

857033

Fig. 6

##### Etymology.

Epithet derived from the name of the host species, Paeonia×suffruticosa.

**Figure 6.**
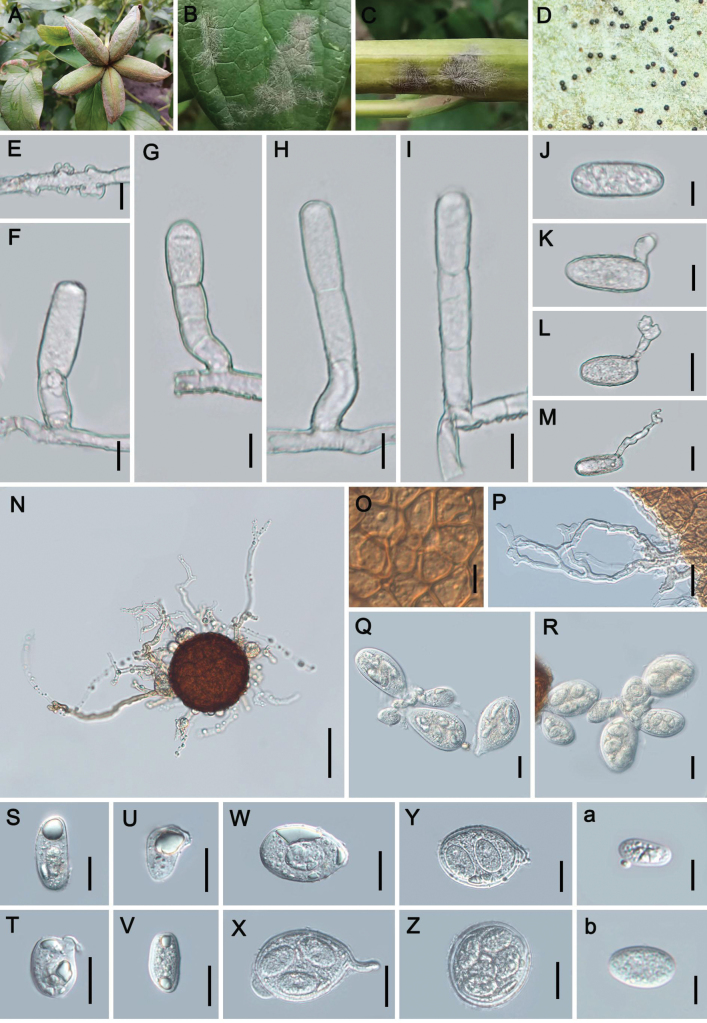
Symptoms and morphology of *Erysiphepaeoniae-suffruticosae* sp. nov. on Paeonia×suffruticosa (holotype: HMJAU-PM92255) **A–D** powdery layer and chasmothecia on buds, leaves and stems **E** hyphal appressorium **F–I** conidiophores **J** conidium **K–M** germ tubes **N** chasmothecium **O** peridium cells **P** appendage **Q–Z** asci (**Q, R** two types asci **S–V** micro-asci **W–Z** macro-asci) **a** micro-ascospores **b** macro-ascospores. Scale bars: 10 µm (**E–K, O, a, b**); 20 µm (**L, M, P–Z**); 50 µm (**N**).

##### Diagnosis.

Differs from *E.paeoniae* in having significantly smaller chasmothecia, 78–98(–118) µm diam. vs. (70–)90–125(–135) µm diam., with longer appendages, 0.5–3.3 times as long as the chasmothecial diam. vs. 0.25–1(–2) times, and significantly different asci and ascospores, and by forming a well-supported species clade in phylogenetic ITS+28S+IGS as well as ITS+28S+IGS+*RPB2*+*TUB* analyses in sister position to *E.paeoniae* on *Paeonialactiflora*.

##### Description.

Mycelium on stems, fruits, perianths and leaves, amphigenous, effuse, often covering the entire surface of the leaves, forming mycelial layers of uneven thickness or patches, persistent; hyphae straighten or flexuous, 4–7 µm wide; hyphal appressoria nipple-shaped to moderately lobed, solitary or in opposite pairs, often several per hyphal cell. **Asexual morph**: Conidiophores on top of mother cell, (22–)29–69 × 8–12 µm long, foot cells cylindrical, (11–)15–28(–36) × 8–10 µm (without condia), often curved at the base, few erect, followed by 0–2 mostly shorter cells; conidia single, cylindrical, long-cylindrical, doliiform, 25–48 × 12–19 µm, length/width ratio 1.4–3.8; germ tubes terminal, septate, partly forming curved aerial germ tubes, tips irregularly forked, partly long-clavate, or medium-long, swollen to club-shaped or lobed. **Sexual morph**: Chasmothecia scattered, produced first on the abaxial surface of the leaf and more numerous than on the adaxial surface, black-brown, hemispherical, 78–98(–118) µm diam.; peridium cells irregularly polygonal, 6–15 µm diam.; appendages 15–28, mycelioid, 1–2 times bifurcately branched, rarely unbranched, also forming small irregular branches or small protuberances, often 1–2 times irregularly branched apically, some small branches tending to be parallel, 0.5–3.3 times as long as the chasmothecial diam., 56–218(–257) µm, 0–1-septate, smooth or rough, dark brown or light brown throughout, or only basally brown and upward becoming colorless; asci dimorphic, micro- and macro-asci mixed together in the same ascoma mostly, 3–6 in total, viz., micro-asci, ovoid, saccate, irregularly ovoid, subglobose or elongate, (14–)20–49 × (11–)18–30 µm, long-stalked, short-stalked to sessile, containing 0–1 spore, ovoid, 10–16 × 9–13 µm; macro-asci, ovoid, ellipsoid-ovoid, subglobose, saccate, 34–65(–69) × 19–45(–51) µm, long-stalked, short-stalked to sessile, containing 1–5-spores, ascospores ovoid, ellipsoid-ovoid, 18–27 × 11–19 µm, colorless.

##### Holotype.

China, Shaanxi Prov. • 1; Yan’an City; 36°37'10"n, 109°27'26"e; ca. 930 m a.s.l.; 27 Sep. 2018; Shu-Rong Tang & Li Liu leg.; on Paeonia×suffruticosa; HMJAU-PM92255. Isotype: same data as for holotype; HMAS 353412.

##### Host.

Paeonia×suffruticosa (Paeoniaceae).

##### Distribution.

Asia (China and South Korea).

##### Additional material examined.

China, Gansu Prov. • 1; Lanzhou City; 36°2'49"n, 103°51'14"e; ca. 1461 m a.s.l.; 20 Sep. 2018; Shu-Rong Tang & Li Liu leg.; on P.×suffruticosa; HMJAU-PM92256. • 1; Lanzhou City; 36°4'13"n, 103°48'50"e; ca. 1560 m a.s.l.; 16 Oct. 2023; Zhao-Yang Zhang & Li Liu leg.; on P.×suffruticosa; HMJAU-PM92254. –Heilongjiang Prov. • 1; Mudanjiang City; 44°35'9"n, 129°37'11"e; ca. 240 m a.s.l.; Fen-Yun Zhao, Vanninh Nguyen, Jing-Sheng Lu & Jia-Ni Li leg.; on P.×suffruticosa; HMJAU-PM92272. –Jilin Prov. • 1; Changchun City; 43°48'21"n, 125°24'17"e; ca. 1460 m a.s.l., 10 Aug. 2023; Zhao-Yang Zhang leg.; on P.×suffruticosa; HMJAU-PM92257.

##### Notes.

Powdery mildew on *Paeonia×suffruticosa* was first found in South Korea and identified as *Erysiphepaeoniae* through combined morphology and ITS sequence analyses (La et al. 2015). Subsequent reports from China confirmed this identification using ITS data (Qian et al. 2016). While the anamorphic morphologies of *E.paeoniae* on P.×suffruticosa (designated *E.paeoniae-suffruticosae*) and *P.lactiflora* (on *E.paeoniae*) show no significant differences, their chasmothecium structures exhibit marked morphological divergence. Notably, *E.paeoniae-suffruticosae* possesses two distinct types of asci and ascospores, whereas *E.paeoniae* exhibits only a single type. The holotype of *E.paeoniae* (hosted on *P.obovata* preserved at the HMAS, Beijing) underwent morphological re-evaluation and molecular analysis; however, all sequencing attempts failed, probably due to the age of the specimen. Despite this limitation, the morphological similarity observed between *E.paeoniae* populations on *P.obovata* and *P.lactiflora* supports the authenticity of *E.paeoniae* on its type host. Significant morphological differentiation between *E.paeoniae-suffruticosae* and *E.paeoniae* is supported by phylogenetic analyses using ITS+28S+IGS, which together support recognition of *E.paeoniae-suffruticosae* as a distinct taxon on *P.×suffruticosa* (Suppl. material 1). Furthermore, in the multi-gene analysis (ITS+28S+IGS+*RPB2*+*TUB*), *E.paeoniae-suffruticosae* formed a distinct clade, providing additional evidence for its taxonomic distinction (Fig. 2).

Field surveys indicate high susceptibility of *P.×suffruticosa* to powdery mildew, suggesting potential broader geographic distribution of *E.paeoniae-suffruticosae* within China.

###### ﻿New records

#### ﻿ Erysiphe malvae

Taxon classificationFungiHelotialesErysiphaceae

Heluta, Ukrayins’k. Bot. Zhurn. 47(4): 75, 1990.

71EB6B32-1A08-5B1C-9450-74814BB9F431

Fig. 7

##### Description.

Mycelium on leaves and stems, effuse or in irregular powdery layers, white, persistent; hyphae 3–8 μm wide; hyphal appressoria nipple-shaped to lobed. **Asexual morph**: Conidiophores erect, sometimes slightly flexuous, 62–116(–133) × 5–9 μm (without conidia), foot cells cylindrical, straight, 33–69 × 5–9 μm, followed by 1–2 cells, significantly shorter or longer than the foot cells, sometimes about as long as foot cells; conidia single, cylindrical, ellipsoid-cylindrical, 25–42(–46) × 12–18 μm, length/width ratio 1.6–4.0, colorless; the germ tube subterminal, medium length, club-shaped, apex simple or somewhat bent and swollen.

**Figure 7.**
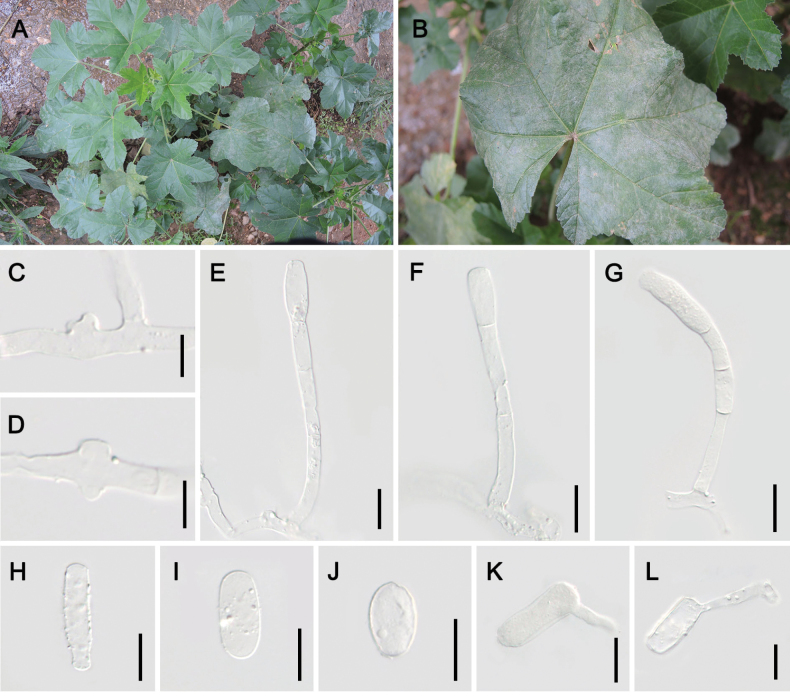
Symptoms and asexual morph of *Erysiphemalvae* on *Malvapusilla* (HMJAU-PM92233) **A, B** powder layer on leaves **C, D** hyphal appressoria **E–G** conidiophores **H–J** conidia **K, L** germ tubes. Scale bars: 10 µm (**C, D**); 20 µm (**E–L**).

##### Host.

*Malvapusilla* (Malvaceae).

##### Distribution.

Asia (China, Iran and Israel), Europe (Ukraine).

##### Additional material examined.

China, Yunnan Prov. • 1; Kunming City; 25°6'51"n, 102°45'9"e; ca. 1890 m a.s.l.; 26 Jun. 2019; Shu-Rong Tang & Jing Feng leg.; on *M.pusilla*; HMJAU-PM92233. • 1; Kunming City; 25°3'27"n, 102°41'55"e; alt. 1920 m a.s.l.; 27 Jun. 2019; Shu-Rong Tang & Jing Feng leg.; HMJAU-PM92234.

##### Notes.

*Erysiphemalvae* was initially described from Europe (Ukraine) and subsequently documented in Asia (Iran and Israel), with its most recent record in Nepal (Adhikari 2021). This represents the first confirmed occurrence of this pathogen in China, where the specimen was collected in Yunnan Province in 2019. Morphological comparison of the Chinese anamorphs revealed fundamental congruence with the original description by Braun and Cook (2012), except for a discrepancy in subsequent cell count: our specimens exhibited 1–2 cells following the foot cells, compared to the 1–3 cells reported in the type description.

Sequence-based verification confirmed the taxonomic identity with *E.malvae* (on *Malvapusilla*), with its phylogenetic position within the *E.betae*/*E.heraclei*/*E.malvae* complex clade (Figs 1, 2).

#### ﻿ Erysiphe punicae

Taxon classificationFungiHelotialesErysiphaceae

T.M. Achundov, Novosti Sist. Nizsh. Rast. 24: 95, 1987.

BBD8D633-AF0F-5C88-833B-38ED8043168F

Fig. 8

##### Description.

Mycelium epiphyllous, thin, in grayish white patches or effuse, later covering entire leaves, persistent; hyphae 3–4 μm wide; hyphal appressoria almost nipple-shaped, slightly lobed, usually in opposite pairs. **Asexual morph**: Conidiophores arising from the upper surface of the mother cells, erect, occasionally slightly curved in the center, about 64–108 × 4–7 μm long, foot-cells cylindrical, straight, occasionally slightly flexuous-sinuous, 29–53(–78) × 4–7 μm, followed by 1–2 usually shorter cells, significantly shorter than foot cells; conidia single, narrowly cylindrical, 21–36(–49) × 6–13 μm, length/width ratio 1.9–4.3, germ tubes subterminal, short, with swollen tips or lobed appressoria.

**Figure 8.**
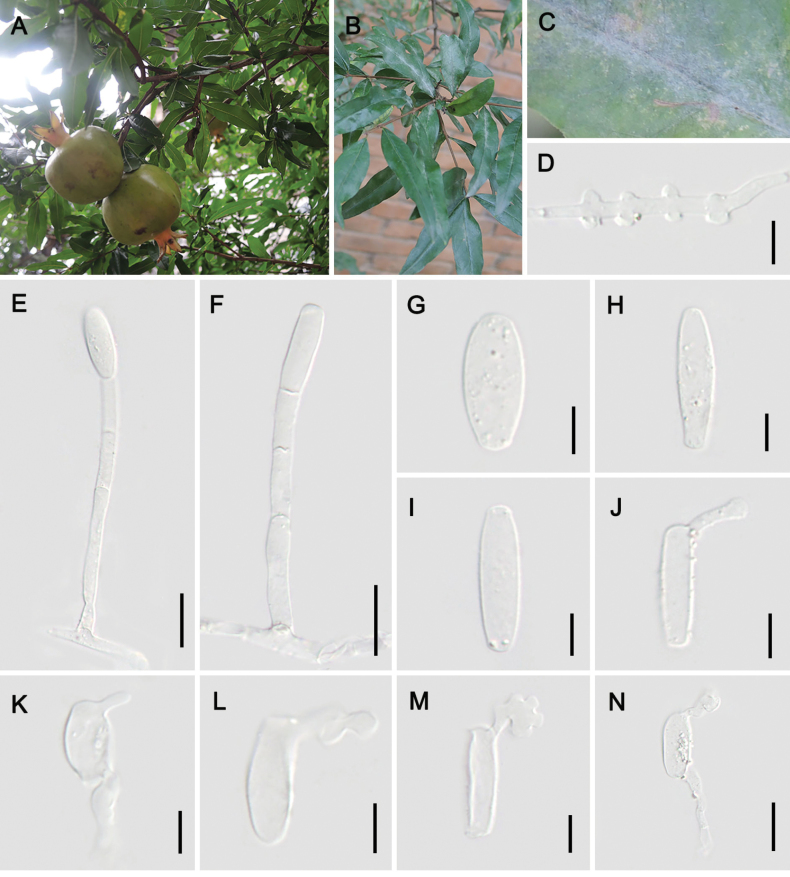
Symptom and asexual morph of *Erysiphepunicae* on *Punicagranatum* (HMJAU-PM92219) **A–C** powdery layer on the leaves **D** hyphal appressoria **E, F** conidiophores **G–I** conidia **J–N** germ tubes. Scale bars: 10 µm (**D, G–M**); 20 µm (**E, F, N**).

##### Host.

*Punicagranatum* (Lythraceae).

##### Distribution.

Asia (China and Iran), Caucasus (Azerbaijan) and Europe (Montenegro).

##### Material examined.

China, Yunnan Prov. • 1; Kunming City; 25°3'30"n, 102°41'43"e; ca. 1900 m a.s.l.; 27 Jun. 2019, Shu-Rong Tang & Jing Feng leg.; on *P.granatum*; HMJAU-PM92219. • 1; same locality; 11 Nov. 2019, Zhao-Yang Zhang & Dan-Ni Jin leg.; on *P.granatum*; HMJAU-PM92220.

##### Notes.

Nearly all historical records of *Erysiphepunicae* are based on anamorphs (Amano 1986), with only one specimen with chasmothecia identified in Montenegro (Braun and Cook 2012), which has been sequenced (ITS+28S) and confirmed the position of this species within the *E.aquilegiae* complex (Bradshaw et al. 2023c). However, systematic verification remains critical as many anamorphic records may represent cryptic species or misidentifications within the complex. In 2023, *E.punicae* was newly documented on pomegranate (*Punicagranatum*) in India by ITS sequence analysis (Watpade et al. 2023). Our phylogenetically verified collections from Yunnan and Hebei provinces, China, showed morphological congruence with *E.punicae* (HAL 2441F) (Bolay et al. 2021) and were supported by phylogenetic analyses (Figs 1, 2). All *E.punicae* sequences were consistently resolved as a distinct, well-supported clade within the *E.aquilegiae* complex clade, confirming its taxonomic validity. This is the first reported occurrence of *E.punicae* in China, extending its known geographic range by both morphological and molecular evidence.

## ﻿Discussion

The discovery of multiple novel *Erysiphe* species in China represents a significant expansion of our understanding of fungal diversity within this genus and its associated plant hosts. These findings include both previously unrecognized cryptic taxa on established host genera (e.g., *Clematis*, *Limonium*) and newly identified pathogens on non-traditional hosts (Paeonia×suffruticosa). In addition, *E.malvae* and *E.punicae* have been newly discovered in China. All new species and the new records are based on the present phylogenetic analyses and tend to reflect a strong co-evolutionary relationship between species of the Erysiphaceae and their hosts. *E.limoniicola* is a newly described species parasitizing plants in the genus *Limonium* (Plumbaginaceae). An unidentified powdery mildew fungus, previously recorded on *L.tetragonum* in Japan (Meeboon et al. 2021), has been confirmed as the new species *E.limoniicola*. *E.paeoniae-suffruticosae* is a new species confined to Paeonia×suffruticosa. Previously, *E.paeoniae* was reported to parasitize a wide range of plants in the genus *Paeonia* and had a broad distribution. La et al. (2015) recorded P.×suffruticosa as a host of *E.paeoniae*, and this species is now classified under the new species *E.paeoniae-suffruticosae*. Additionally, there may be other cryptic species on *Paeonia*, as demonstrated in Fig. 1, where the powdery mildew fungi on *Paeonia* [P.coriaceavar.atlantica and P.dauricasubsp.wittmanniana (= *P.wittmanniana*)] originating from Switzerland form distinct clades.

Another one, *Erysipheclematidis* is confined to *Clematis* spp. (Ranunculaceae) in the northwestern regions (Gansu, Qinghai and Shaanxi provinces) of China. Previously, powdery mildews on *Clematis* were all described as *E.aquilegiae* (Braun and Cook 2012), including the *Clematis* specimen we collected in Jilin Province (HMJAU-PM92189). Both rDNA sequence and multilocus sequence phylogenetic analyses show that *E.clematidis* forms a separate branch with high support (Figs 1, 2, Suppl. material 1). However, in the ITS+28S and ITS+28S+IGS phylogenetic trees, *E.clematidis* formed a sister clade to *E.aquilegiae*s. lat., whereas in the multi-locus phylogeny (ITS+28S+IGS+*RPB2*+*TUB*), it clustered as a sister clade to the lineage containing *E.acetosae*, *E.betae*, *E.heraclei*, *E.howeana*, *E.limonii*, *E.limoniicola*, *E.malvae*, *E.paeoniae*, *E.paeoniae-suffruticosae*, *E.polygoni* and *E.rumicicola*.. The systematic relationship between *E.clematidis* and the *E.aquilegiae* complex needs to be further explored. This newly recognized species provides a crucial perspective for evolutionary studies. Its distinct phylogenetic position relative to the *E.aquilegiae* complex makes it an ideal outgroup for analyzing the evolutionary dynamics within this complex. Additionally, the phylogenetic position of *E.actinostemmatis* was first definitively established in this study based on the obtained sequence data, which formed a sister group to the subclade containing *E.hommae*, *E.neolycopersici*, *E.pileae*, and *E.sedi* within the *E.aquilegiae* complex clade. The foundational ITS phylogenetic framework by Takamatsu et al. (2015a) first delineated the *E.aquilegiae* clade (complex), which encompassed numerous taxa including *E.catalpae*, *E.euphorbiae*, *E.macleayae*, *E.knautiae*, *E.coriariae*, *E.circaeae*, *E.sedi*, *E.hommae*, *E.chloranthi*, *E.pileae*, *E.takamatsui*, *E.neolycopersici*, *Ps.hortensiae*, and *Ps.boroniae*. Subsequent studies by Shin et al. (2019) and Bradshaw et al. (2020b) further explored this complex, establishing criteria for species delimitation. However, while *E.parnassiae*, *E.lythri*, and *E.punicae* cluster within the broader *E.aquilegiae* clade in phylogenetic trees, single-locus analyses (ITS/28S rDNA) have proven inadequate for resolving species-level relationships within the core complex. This limitation prompted Bradshaw et al. (2023c) to employ multilocus approaches (ITS/28S/*CAM*/*GAPDH*/*GS*/*RPB2*/*TUB*), which improved resolution but still revealed persistent ambiguities, many taxa like *E.sedi*, *E.pileae*, and *E.hommae* remain intermingled in subclades. In the multilocus phylogenetic analyses of this study, *E.coriariicola*, *E.knautiae*, and *E.chloranthi* formed well-supported subclades, whereas *E.sedi*, *E.pileae*, *E.hommae* and others exhibited the same intermixing patterns observed in Bradshaw et al. (2023c) (Fig. 2).

Furthermore, currently performed phylogenetic analyses of collections on diverse Ranunculaceae revealed undescribed speciation within *E.aquilegiae* (var.aquilegiae and var.ranunculi) (Figs 1, 2, Suppl. material 1). *E.aquilegiae* as previously circumscribed (Braun and Cook 2012) potentially comprises more different species, viz., E.aquilegiaevar.ranunculi on *Ranunculus* spp. and *Clematisflorida*, E.aquilegiaevar.aquilegiae on *Aquilegiavulgaris*, and one or more undescribed species (*Erysiphe* spp.) on *Anemonastrumcanadense* comb. nov. (= *Anemonecanadensis*) (FH01122081) (Monsyakin 2016), *Aquilegiacoerulea* (FH00941236), *Clematis* [*Cl.stans* (MUMHJPN 0293) and *Cl.montana* (MA51 and MA52)], *Thalictrum* [*T.delavayi* (FH01131052), and *T.revolutum* (FH01131051) (Figs 1, 2, Suppl. material 1). These results indicate that, although multilocus analyses were used, there are still many taxa with identical or almost identical sequences. Since the *E.aquilegiae* complex clade is very large and contains many species with a global distribution and a wide host range, more comprehensive analyses of extensive collections from diverse geographical regions and hosts from around the world are needed to better understand this complex.

Phylogenetic analyses incorporating powdery mildew sequences from two Polygonaceae genera (*Polygonum*, and *Rumex*) have further clarified the species of powdery mildews associated with Polygonaceae in China. Our expanded phylogenetic framework incorporating *E.rumicicola* (Darsaraei et al. 2023) and *E.acetosae* (Bradshaw et al. 2024b) from *Rumex* provides new insights into Chinese *Rumex*-infesting powdery mildews, confirming *E.rumicicola* as the resident pathogen. It also indicates that the powdery mildew on specimens on the *Rumex* genus from South Korea (KACC 30382, KUS F30682, and KUS F30366) is the same species as that found in China, *E.rumicicola*, rather than *E.polygoni* (Fig. 1). Another species on Polygonaceae, *E.bistortae* on *Bistortaamplexicaulis* (Zafar et al. 2023), belongs to the *E.betae*/*E.heraclei*/*E.malvae* complex clade. These species exhibit minimal genetic differentiation based on ITS sequences and are primarily distinguished from other members of the *E.betae*/*E.heraclei*/*E.malvae* complex clade, including the newly recorded species *E.malvae* introduced in this study, through biological and morphological characteristics. This further highlights the limitations of rDNA sequence-based phylogenetics in this group.

Based on current trends, many fungal species have relatively broad host ranges, considering environmental and ecological factors (Phukhamsakda et al. 2020). Powdery mildews also have the ability to switch between different host plants. This host-switching capability may further lead to speciation, especially when there are changes in the ecological niches of host plants or under conditions of geographical isolation. Therefore, the evolution and speciation processes of powdery mildews are likely to be closely related to the diversity of host plants and their ecological niches. In terms of phylogenetic analysis, our research results indicate that, in addition to the sequences from traditional gene regions such as *RPB2* and *TUB*, IGS rDNA sequences also hold significant potential to enhance the resolution of phylogenetic analyses of sect. Erysiphe species. This is particularly important in complexes that are poorly resolved based solely on ITS+28S sequences. By integrating these different gene regions, we can gain a more comprehensive understanding of the evolutionary history and speciation processes of powdery mildews.

## ﻿Conclusions

Three new species and two new records of Erysiphesect.Erysiphe from China were described and illustrated. These five new taxa are well supported by molecular phylogenetic data and morphological evidence. This study provides support for exploring the evolutionary relationship between parasitic fungi and their host plants, not only by conducting a multi-gene phylogenetic analysis that combines the *RPB2* and *TUB* regions, but also by further validating that the molecular marker (IGS rDNA) is applicable for distinguishing species within sect. Erysiphe . The results enrich the morphology, hosts, and distribution of powdery mildew fungi, and provide new insights into phylogenetic and taxonomic studies of powdery mildew fungi.

## Supplementary Material

XML Treatment for Erysiphe clematidis

XML Treatment for Erysiphe limoniicola

XML Treatment for Erysiphe paeoniae-suffruticosae

XML Treatment for Erysiphe malvae

XML Treatment for Erysiphe punicae

## ﻿Additional information

### Author ORCIDs

Zhao-Yang Zhang https://orcid.org/0009-0002-2507-8480

Tie-Zhi Liu https://orcid.org/0000-0003-1751-2195

Li Liu https://orcid.org/0000-0002-6754-3750

Jing Feng https://orcid.org/0000-0001-5229-2648

Tom Hsiang https://orcid.org/0000-0002-9549-1847

Shu-Yan Liu https://orcid.org/0000-0001-9082-1053

### Data availability

All of the data that support the findings of this study are available in the main text or Supplementary Information.

## Supplementary materials

Supplementary material 1 Phylogenetic tree of selected species within sect. Erysiphe based on the ITS+28S+IGS regions Data type pdf Explanation note Bootstrap values greater than 60% for the maximum likelihood (ML) analyses are displayed followed by posterior probabilities ≥ 0.70 of Bayesian inference (BI) analyses. HT = Holotype; the color box indicates new species; bold font indicates the new species obtained in this study; blue bold font indicates new record species. https://binary.pensoft.net/file/1344266This dataset is made available under the Open Database License (http://opendatacommons.org/licenses/odbl/1.0/). The Open Database License (ODbL) is a license agreement intended to allow users to freely share, modify, and use this Dataset while maintaining this same freedom for others, provided that the original source and author(s) are credited.Zhao-Yang Zhang

Supplementary material 2 List of taxa, host, voucher, collection locality and GenBank accession number of the specimens downloaded from NCBI Data type xlsx https://binary.pensoft.net/file/1344267This dataset is made available under the Open Database License (http://opendatacommons.org/licenses/odbl/1.0/). The Open Database License (ODbL) is a license agreement intended to allow users to freely share, modify, and use this Dataset while maintaining this same freedom for others, provided that the original source and author(s) are credited.Zhao-Yang Zhang

Supplementary material 3 Alignment file for constructing the ITS+28S phylogenetic tree Data type fas https://binary.pensoft.net/file/1344268This dataset is made available under the Open Database License (http://opendatacommons.org/licenses/odbl/1.0/). The Open Database License (ODbL) is a license agreement intended to allow users to freely share, modify, and use this Dataset while maintaining this same freedom for others, provided that the original source and author(s) are credited.Zhao-Yang Zhang

Supplementary material 4 Alignment file for constructing the ITS+28S+IGS+*RPB2*+*TUB* phylogenetic tree Data type fas https://binary.pensoft.net/file/1344269This dataset is made available under the Open Database License (http://opendatacommons.org/licenses/odbl/1.0/). The Open Database License (ODbL) is a license agreement intended to allow users to freely share, modify, and use this Dataset while maintaining this same freedom for others, provided that the original source and author(s) are credited.Zhao-Yang Zhang
