# Supplementary material for: ﻿Discover hidden taxa of Erysiphe section Erysiphe fungi (Ascomycota, Erysiphaceae) based on morphology and multilocus phylogeny in China
Source: MycoKeys. 2025 Jun 4;118:119–46. doi: 10.3897/mycokeys.118.154217 (PMC12159665; doi:10.3897/mycokeys.118.154217)
Supplement: Supplementary material 1 — Phylogenetic tree of selected species within sect. Erysiphe based on the ITS+28S+IGS regions [file mycokeys-118-119-s001.pdf]

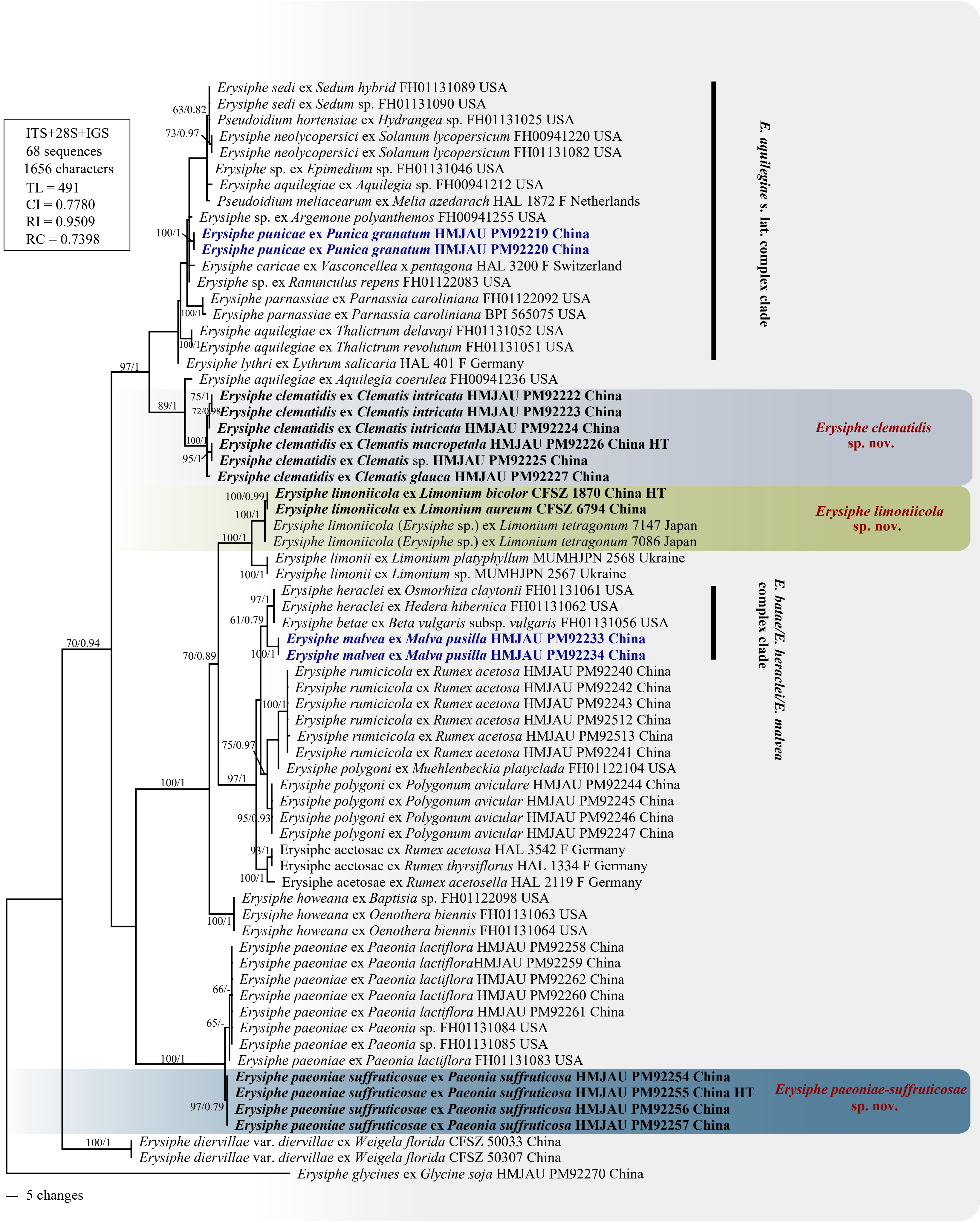

ITS+28S+IGS
68 sequences
1656 characters
TL = 491
CI = 0.7780
RI = 0.9509
RC = 0.7398
Erysiphe sedi ex Sedum hybrid FH01131089 USA
Erysiphe sedi ex Sedum sp. FH01131090 USA
Pseudoidium hortensiae ex Hydrangea sp. FH01131025 USA
Erysiphe neolycopersici ex Solanum lycopersicum FH00941220 USA
Erysiphe neolycopersici ex Solanum lycopersicum FH01131082 USA
Erysiphe sp. ex Epimedium sp. FH01131046 USA
Erysiphe aquilegiae ex Aquilegia sp. FH00941212 USA
Pseudoidium meliacearum ex Melia azedarach HAL 1872 F Netherlands
Erysiphe sp. ex Argemone polyanthemos FH00941255 USA
Erysiphe punicae ex Punica granatum HMJAU PM92219 China
Erysiphe punicae ex Punica granatum HMJAU PM92220 China
Erysiphe caricae ex Vasconcellea x pentagona HAL 3200 F Switzerland
Erysiphe sp. ex Ranunculus repens FH01122083 USA
Erysiphe parnassiae ex Parnassia caroliniana FH01122092 USA
Erysiphe parnassiae ex Parnassia caroliniana BPI 565075 USA
Erysiphe aquilegiae ex Thalictrum delavayi FH01131052 USA
Erysiphe aquilegiae ex Thalictrum revolutum FH01131051 USA
Erysiphe lythri ex Lythrum salicaria HAL 401 F Germany
Erysiphe aquilegiae ex Aquilegia coerulea FH00941236 USA
Erysiphe clematidis ex Clematis intricata HMJAU PM92222 China
Erysiphe clematidis ex Clematis intricata HMJAU PM92223 China
Erysiphe clematidis ex Clematis intricata HMJAU PM92224 China
Erysiphe clematidis ex Clematis macropetala HMJAU PM92226 China HT
Erysiphe clematidis ex Clematis sp. HMJAU PM92225 China
Erysiphe clematidis ex Clematis glauca HMJAU PM92227 China
Erysiphe limoniicola ex Limonium bicolor CFSZ 1870 China HT
Erysiphe limoniicola ex Limonium aureum CFSZ 6794 China
Erysiphe limoniicola (Erysiphe sp.) ex Limonium tetragonum 7147 Japan
Erysiphe limoniicola (Erysiphe sp.) ex Limonium tetragonum 7086 Japan
Erysiphe limonii ex Limonium platyphyllum MUMHJPN 2568 Ukraine
Erysiphe limonii ex Limonium sp. MUMHJPN 2567 Ukraine
Erysiphe heraclei ex Osmorhiza claytonii FH01131061 USA
Erysiphe heraclei ex Hedera hibernica FH01131062 USA
Erysiphe betae ex Beta vulgaris subsp. vulgaris FH01131056 USA
Erysiphe malvea ex Malva pusilla HMJAU PM92233 China
Erysiphe malvea ex Malva pusilla HMJAU PM92234 China
Erysiphe rumicicola ex Rumex acetosa HMJAU PM92240 China
Erysiphe rumicicola ex Rumex acetosa HMJAU PM92242 China
Erysiphe rumicicola ex Rumex acetosa HMJAU PM92243 China
Erysiphe rumicicola ex Rumex acetosa HMJAU PM92512 China
Erysiphe rumicicola ex Rumex acetosa HMJAU PM92513 China
Erysiphe rumicicola ex Rumex acetosa HMJAU PM92241 China
Erysiphe polygoni ex Muehlenbeckia platyclada FH01122104 USA
Erysiphe polygoni ex Polygonum aviculare HMJAU PM92244 China
Erysiphe polygoni ex Polygonum avicular HMJAU PM92245 China
Erysiphe polygoni ex Polygonum avicular HMJAU PM92246 China
Erysiphe polygoni ex Polygonum avicular HMJAU PM92247 China
Erysiphe acetosae ex Rumex acetosa HAL 3542 F Germany
Erysiphe acetosae ex Rumex thyrsiflorus HAL 1334 F Germany
Erysiphe acetosae ex Rumex acetosella HAL 2119 F Germany
Erysiphe howeana ex Baptisia sp. FH01122098 USA
Erysiphe howeana ex Oenothera biennis FH01131063 USA
Erysiphe howeana ex Oenothera biennis FH01131064 USA
Erysiphe paeoniae ex Paeonia lactiflora HMJAU PM92258 China
Erysiphe paeoniae ex Paeonia lactifloraHMJAU PM92259 China
Erysiphe paeoniae ex Paeonia lactiflora HMJAU PM92262 China
Erysiphe paeoniae ex Paeonia lactiflora HMJAU PM92260 China
Erysiphe paeoniae ex Paeonia lactiflora HMJAU PM92261 China
Erysiphe paeoniae ex Paeonia sp. FH01131084 USA
Erysiphe paeoniae ex Paeonia sp. FH01131085 USA
Erysiphe paeoniae ex Paeonia lactiflora FH01131083 USA
Erysiphe paeoniae suffruticosae ex Paeonia suffruticosa HMJAU PM92254 China
Erysiphe paeoniae suffruticosae ex Paeonia suffruticosa HMJAU PM92255 China HT
Erysiphe paeoniae suffruticosae ex Paeonia suffruticosa HMJAU PM92256 China
Erysiphe paeoniae suffruticosae ex Paeonia suffruticosa HMJAU PM92257 China
Erysiphe diervillae var. diervillae ex Weigela florida CFSZ 50033 China
Erysiphe diervillae var. diervillae ex Weigela florida CFSZ 50307 China
Erysiphe glycines ex Glycine soja HMJAU PM92270 China
E. aquilegiae s. lat. complex clade
Erysiphe clematidis sp. nov.
Erysiphe limoniicola sp. nov.
E. batae/E. heraclei/E. malvea complex clade
Erysiphe paeoniae-suffruticosae sp. nov.
63/0.82
73/0.97
100/1
100/1
100/1
97/1
89/1
75/1
72/0.98
100/1
95/1
100/0.99
100/1
100/1
100/1
97/1
61/0.79
100/1
70/0.94
70/0.89
100/1
75/0.97
97/1
100/1
95/0.93
93/1
100/1
100/1
66/-
65/-
100/1
97/0.79
100/1
5 changes
